# Molecular and structural basis of an ATPase-nuclease dual-enzyme anti-phage defense complex

**DOI:** 10.1038/s41422-024-00981-w

**Published:** 2024-06-04

**Authors:** Qiyin An, Yong Wang, Zhenhua Tian, Jie Han, Jinyue Li, Fumeng Liao, Feiyang Yu, Haiyan Zhao, Yancheng Wen, Heng Zhang, Zengqin Deng

**Affiliations:** 1 Key Laboratory of Virology and Biosafety, Wuhan Institute of Virology, Chinese Academy of Sciences, Wuhan, Hubei China; 2 University of Chinese Academy of Sciences, Beijing, China; 3 Department of Human Anatomy, School of Basic Medical Sciences, Tianjin Medical University, Tianjin, China; 4 Department of Biochemistry and Molecular Biology, School of Basic Medical Sciences, Tianjin Medical University, Tianjin, China; 5 State Key Laboratory of Virology, College of Life Sciences, Wuhan University, Wuhan, Hubei China; 6 Key Laboratory of Gastrointestinal Cancer (Fujian Medical University), Ministry of Education, Fuzhou, Fujian China; 7 Hubei Jiangxia Laboratory, Wuhan, Hubei China

**Keywords:** Cryoelectron microscopy, Toll-like receptors

## Abstract

Coupling distinct enzymatic effectors emerges as an efficient strategy for defense against phage infection in bacterial immune responses, such as the widely studied nuclease and cyclase activities in the type III CRISPR-Cas system. However, concerted enzymatic activities in other bacterial defense systems are poorly understood. Here, we biochemically and structurally characterize a two-component defense system DUF4297–HerA, demonstrating that DUF4297–HerA confers resistance against phage infection by cooperatively cleaving dsDNA and hydrolyzing ATP. DUF4297 alone forms a dimer, and HerA alone exists as a nonplanar split spiral hexamer, both of which exhibit extremely low enzymatic activity. Interestingly, DUF4297 and HerA assemble into an approximately 1 MDa supramolecular complex, where two layers of DUF4297 (6 DUF4297 molecules per layer) linked via inter-layer dimerization of neighboring DUF4297 molecules are stacked on top of the HerA hexamer. Importantly, the complex assembly promotes dimerization of DUF4297 molecules in the upper layer and enables a transition of HerA from a nonplanar hexamer to a planar hexamer, thus activating their respective enzymatic activities to abrogate phage infection. Together, our findings not only characterize a novel dual-enzyme anti-phage defense system, but also reveal a unique activation mechanism by cooperative complex assembly in bacterial immunity.

## Introduction

Bacteriophages (phages) that infect bacteria and archaea (prokaryotes) are the most abundant and diverse biological entities on earth, with an estimated total abundance of 10^31^.^1,2^ Prokaryotes and phages are in an evolutionary arms race, leading to the emergence of multiple anti-phage defense systems that mitigate phage infection. Among them, adaptive immune system CRISPR-Cas and innate immune system restriction-modification are the two most canonical and widely distributed host defense systems and are well characterized.^3,4^ Bioinformatic analysis and following experimental validation have explored many new types of systems that complement the activities of canonical defense systems, providing multi-layered host defense.^5–11^ However, the mechanisms of these newly identified defense systems remain yet to be well explored.

Degrading viral genetic materials is the most prevalent immune strategy mediated by enzymatic effectors in diverse immune systems. The adaptive CRISPR-Cas immune systems and the restriction-modification systems target foreign nucleic acids in a site-specific manner.^12^ In contrast, the cyclic oligonucleotide-based antiviral signaling system (CBASS) and nucleotide-binding leucine-rich repeat (NLR)-like proteins can degrade both phage and host DNA to abrogate viral replication.^13,14^ The cellular essential metabolites have also emerged as common targets of host-directed immune responses that arrest cell growth to impair viral replication. For instance, the human SAMHD1 diminishes infection of retroviruses and herpesviruses by depleting intracellular deoxynucleotide triphosphates (dNTPs).^15^ Analogously, the NADase domain of short prokaryotic Argonaute systems degrades NAD^+^,^11,16,17^ and the CBASS immune effector Cap17 cleaves ATP, which then triggers cell death or arrest cell growth to limit phage propagation.^18^ While most immune systems employ one type of immune strategy to exert antiviral function, only limited systems comprise multiple components associated with enzymatic activities targeting two or more substrates. For example, a recently discovered type III-E CRISPR-Cas system possesses a sequence-specific ribonuclease activity to degrade viral RNA and a programmable RNA-activated proteolytic activity to cleave a sigma factor inhibitor.^19–22^

Most recently, an intriguing two-gene (*DUF4297* and *HerA*, respectively) anti-phage defense system has been identified.^6^ *DUF4297* encodes a protein containing an N-terminal DUF4297 domain (a putative PD-(D/E)xK-family nuclease) with predicted yet-to-be biochemically characterized nuclease activity and a C-terminal domain (CTD) with unknown function. *HerA*, widely distributed in archaea and bacteria, encodes a DNA motor protein, which can hydrolyze ATP and translocate along DNA. Heterologous expression of the *DUF4297–HerA* operon in *Escherichia coli* K-12 MG1655 has rendered the host strong defense against dsDNA phages P1 and λ and relatively weak defense against dsDNA phages T3, T4, and T7.^6^ Mutating the predicted nuclease and ATPase activities-associated residues dismissed the anti-phage activity, indicating that the predicted nuclease and ATPase activities are indispensable for anti-phage defense. The DUF4297–HerA system may represent immune systems combining the two major immune strategies mentioned above to exert antiviral function. However, the lack of biochemical data to characterize the natural features of the enzymatic activities and detailed structural information of this system limits our mechanistic understanding of this novel immune system.

In this study, we found that DUF4297 and HerA form a stable giant complex and determined a series of high-resolution cryo-EM structures of HerA alone and DUF4297–HerA complex: a HerA alone structure (3.14 Å), a DUF4297–HerA complex structure (2.87 Å), DUF4297–HerA in complex with AMPPNP and DNA substrates in two functional states (2.73 Å and 2.92 Å) and a DUF4297–HerA in complex with ATPγS and DNA substrates (2.81 Å). We further biochemically characterized that DUF4297–HerA giant complex possesses promiscuous dsDNA nuclease and ATPase activities attributed to DUF4297 and HerA, respectively. By contrast, both DUF4297 alone and HerA alone exhibit extremely low enzymatic activity. Combining structure-guided mutagenesis and biochemical and functional studies, we unravel novel and interesting mechanisms of supramolecular assembly and regulation of a dual enzyme anti-phage immune system.

## Results

### HerA alone exists as a split spiral hexamer

To investigate the molecular mechanism underlying the DUF4297–HerA immune system, we expressed DUF4297 and HerA in *E. coli* BL21(DE3) and purified the individual protein to homogeneity by affinity chromatography and size-exclusion chromatography. The elution volume on gel filtration and SDS-PAGE gel results indicate that both DUF4297 alone and HerA alone form oligomers in solution (Supplementary information, Fig. S1a, b). Further analytical ultracentrifugation shows that DUF4297 exists as a dimer (Supplementary information, Fig. S1e). We also co-expressed DUF4297 and HerA and purified the complex to homogeneity. The elution volume on gel filtration and SDS-PAGE gel results suggest that DUF4297 and HerA form a stable complex (Supplementary information, Fig. S1c).

**Fig. 1:**
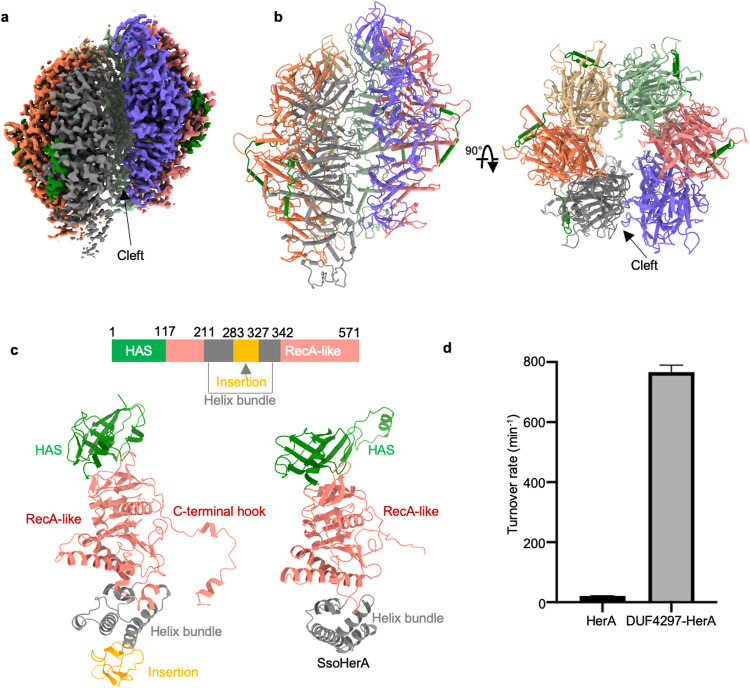
Cryo-EM structure of HerA hexamer. **a** Cryo-EM density map of HerA. **b** Overall structure of HerA. **c** Domain organization of HerA. Each domain is labeled and in a different color. The structure of *Sso*HerA (PDB: 4D2I) is shown for comparison. **d** ATPase activities of HerA and DUF4297–HerA complex.

We first determined the cryo-EM structure of HerA alone at an overall resolution of 3.14 Å. The high quality of the cryo-EM density map allowed us to build the nearly full-length model of HerA (Fig. 1a, b; Supplementary information, Fig. S2a–e). The single HerA molecule adopts an elongated structure. It can be divided into three domains from top to bottom: an N-terminal β-barrel domain, also named HAS, which is a characteristic β-barrel fold seen at the bovine F1-ATP synthase and HerA,^23,24^ the central RecA-like catalytic core (RecA-like), and a flanking four-helix bundle (Helix bundle) (Fig. 1c). This domain organization closely resembles the thermophilic archaeon *Sulfolobus solfataricus* HerA (*Sso*HerA), which shares 26% sequence identity with HerA in the DUF4297–HerA system.^24^ Compared with *Sso*HerA, HerA includes an additional insertion in the four-helix bundle and a hook structure at the C-terminal end (Fig. 1c). Unexpectedly, HerA assembles into a split spiral hexamer with a cleft between the topmost and bottommost subunits (Fig. 1b; Supplementary information, Fig. S2f). This is in remarkable contrast to previously determined structures of HerA homologs, which form a planar symmetric hexamer.^24–26^ The C-terminal hook docks into the surface grooves of the neighboring subunit to strengthen the HerA oligomerization; however, this interaction is disrupted between the topmost and bottommost subunits, destabilizing the hexamer formation (Supplementary information, Fig. S2f). Consistently, 3D classification reveals that two classes form pentamer with one subunit falling apart from the hexameric HerA (Supplementary information, Fig. S2c). The unexpected split spiral conformation of HerA promotes us to test whether HerA alone can hydrolyze ATP as do its homologs. Interestingly, HerA alone shows extremely low ATPase activity. By contrast, the DUF4297–HerA complex exhibits a high turnover rate under the same testing condition, indicating that HerA alone is in a low-activity state (Fig. 1d).

### DUF4297 and HerA assemble into a supramolecular complex

**Fig. 2:**
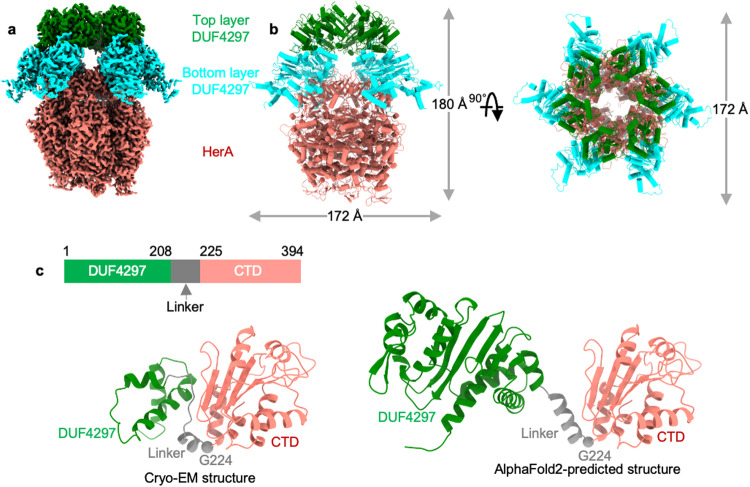
Cryo-EM structure of the DUF4297–HerA complex. **a** Cryo-EM density map of DUF4297–HerA complex. **b** Overall structure of the DUF4297–HerA complex. HerA, the bottom layer DUF4297, and the top layer DUF4297 are colored salmon, cyan, and green, respectively. **c** Domain organization of DUF4297. Each domain is labeled and color-coded. The AlphaFold2-predicted structure of DUF4297 is shown for comparison.

To reveal the assembly mode of this complex, we further determined the cryo-EM structure of DUF4297–HerA complex at an overall resolution of 2.87 Å with C2 symmetry imposed (Supplementary information, Fig. S3). The high quality of the cryo-EM density map allowed us to build the CTD of DUF4297, whereas only a partial part of the N-terminal DUF4297 domain was modeled due to the weak density of this region. Although the densities corresponding to the N-terminal DUF4297 domain were observed at lower thresholds, we could not obtain a meaningful reconstruction of this region after trying different data processing strategies. The cryo-EM structure reveals that twelve DUF4297 molecules and six HerA molecules form a giant assembly with a molecular weight of ~1 MDa. The architecture of the resolved complex has a shape that resembles a human head in a hat, with a 172 Å × 172 Å × 180 Å dimension. The HerA forming a planar hexameric ring resembles the head, while twelve DUF4297 molecules, organized into the upper and the lower layers, resemble the hat (Fig. 2a, b).

The CTD of DUF4297 is composed of a four-stranded parallel β-sheet sandwiched by six helices α1–α6. Helices α1–α3 and α6 are located at the bottom of the β-sheet, while helices α4 and α5 sit on top of the β-sheet (Supplementary information, Fig. S3f). The CTD is connected to the resolved part of the N-terminal DUF4297 domain via a helix with few contacts with other parts of the protein and the boundary is a glycine residue (G224) (Fig. 2c), contributing to the overall conformational plasticity of the full-length protein, particularly the flexible relative positioning of the DUF4297 domain and CTD. Indeed, the resolved part of the DUF4297 domain and the CTD align remarkably well with the respective domains of the AlphaFold2-predicted structure, whereas two full-length structures display different conformations (Supplementary information, Fig. S3f, g).

### Assembly of the DUF4297–HerA complex

**Fig. 3:**
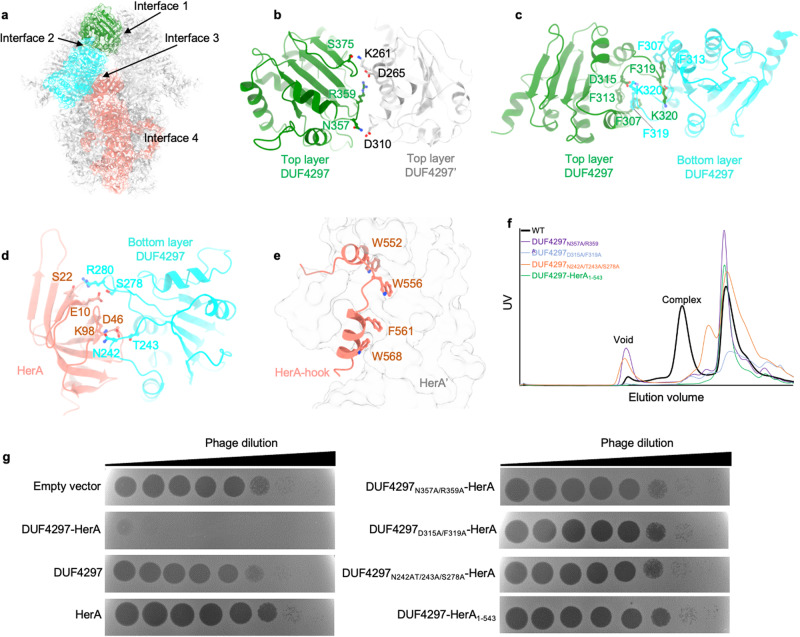
Assembly of DUF4297–HerA complex. **a** Four key assembly interfaces of the DUF4297–HerA complex are indicated. **b** Detailed interactions between the CTDs of two adjacent upper layer DUF4297 molecules. **c** Detailed interactions between the CTDs of the upper layer DUF4297 and the corresponding bottom layer DUF4297. **d** Detailed interactions between the CTD of the bottom layer DUF4297 and the HAS domain of HerA. **e** Detailed interactions between the C-terminal hook and the adjacent HerA. **f** Size-exclusion chromographs of DUF4297–HerA wild type (WT) and mutants harboring mutations at the assembly interface. **g** Plaques of phage λ on cells expressing empty vector, DUF4297–HerA WT, and the indicated mutants. 10-fold serial dilutions of the phage lysate were dropped on the plates.

HerA forms a planar hexameric structure with a central pore in the DUF4297–HerA complex, which is significantly different from the nonplanar conformation of HerA alone. The base of the hexamer, supposed to be the DNA entry site, is closed by the insertion subdomain. The HAS domains and two layers of DUF4297 adopt a nearly C6 symmetric conformation with a continuous channel. The organization of the DUF4297–HerA complex indicates that the nonplanar conformation of HerA alone might not be compatible with complex formation, which was confirmed by the in vitro complex reconstitution assay. No DUF4297–HerA complex peak appeared when purified individual DUF4297 and HerA proteins were mixed in a gel filtration chromatography assay (Supplementary information, Fig. S1d). Six DUF4297 molecules from the upper layer are held together primarily through mixed salt-bridge and hydrogen-bond interactions mediated by the adjacent CTD domains (Fig. 3a, b). Six DUF4297 molecules from the lower layer do not contact each other but form a tail-to-tail dimer with one neighboring DUF4297 from the upper layer through a loop connecting helices α3 and α4. Detailed analysis revealed that hydrophobic interactions dominate the interactions in this interface (Fig. 3c). The tail-to-tail dimer might also be the major conformation of DUF4297 when expressed alone (Supplementary information, Fig. S1e–h). Mutating residues of this dimerization interface dramatically destabilized the protein, as evidenced by the fact that no soluble protein was obtained. The loops connecting helix α1 and β-strand β1, and helix α2 and β-strand β2 of CTD of the lower layer DUF4297 form extensive polar interactions with the HAS domain from HerA (Fig. 3d). Each of the three major segments (HAS, RecA-like, and Helix bundle) makes extensive interactions with the same domain of the adjacent subunit in the HerA hexameric ring. Meanwhile, the C-terminal hook docks into the groove of the neighboring subunit, with four large bulky residues inserted into hydrophobic pockets, further strengthening the interactions (Fig. 3e). The hook is connected to the rest of the protein through a long loop, potentially allowing the hook to maintain a stable interaction even when conformational changes occur among subunits. Mutating residues on these interfaces severely affected the complex formation and impaired the anti-phage activities, further confirming the critical roles of intermolecular contacts for complex assembly and that the complex formation is necessary for anti-phage function (Fig. 3f, g).

### DNA binding by the DUF4297–HerA complex

**Fig. 4:**
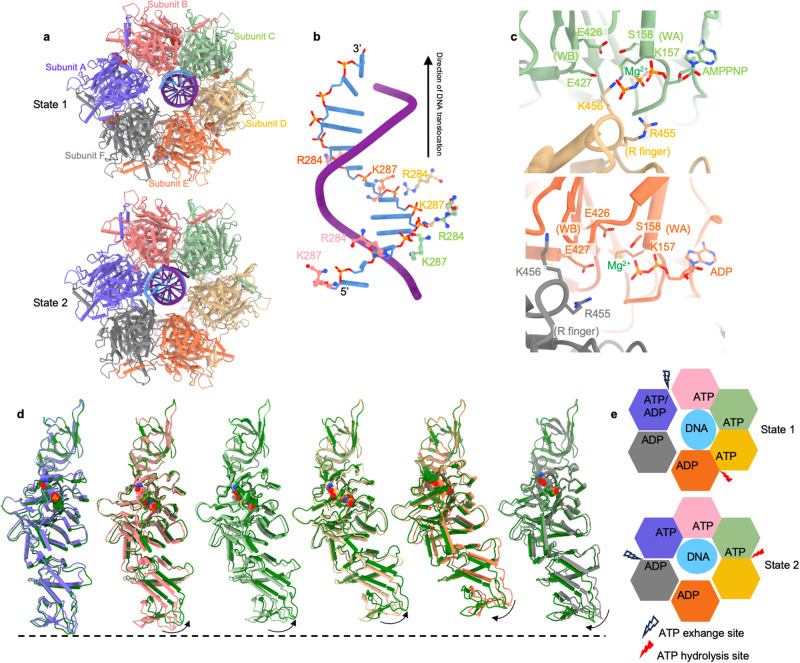
Cryo-EM structures of DUF4297–HerA complexed with ATP analogs and DNA. **a** Bottom view of DUF4297–HerA complexed with ATP analogs and DNA in state 1 (upper panel) and state 2 (lower panel). DUF4297 is removed for clarity. **b** Interactions between DNA and the two basic residues of four DNA-engaged subunits in state 1. **c** Detailed interactions between HerA and the phosphate groups of AMPPNP (upper panel) and ADP (lower panel). **d** Conformational changes of each HerA subunit between state 1 and state 2. The HerA subunit of state 1 is color-coded as in **a**, while the HerA subunit of state 2 is colored green. **e** Nucleotide-binding status in state 1 (upper panel) and state 2 (lower panel).

Given that HerA in the DUF4297–HerA shares a similar structure with its homologs and that HerA homologs were experimentally shown to hydrolyze ATP and translocate along DNA,^24–26^ we speculated that DUF4297–HerA can also translocate along DNA. We incubated a 59-bp synthetic duplex DNA (dsDNA) and ATP analogs (AMPPNP or ATPγS) with purified DUF4297–HerA complex and determined three high-resolution cryo-EM structures. Two distinct classes from the sample with AMPPNP yielded high-resolution reconstructions with overall resolutions of 2.73 Å and 2.92 Å, respectively (Supplementary information, Fig. S4). These two structures differ significantly in their DNA-engaged HerA subunits and adenine nucleotide binding status of each subunit. We referred to these structures as state 1 and state 2, respectively (Fig. 4a). Particles in the sample with ATPγS generated a consensus reconstruction essentially identical to state 1 (Supplementary information, Fig. S5). The DNA densities were well-defined for more than 13 bp, which enters the central pore of the HerA hexameric ring from the open HerA base (Supplementary information, Figs. S4g, h, S5h). The six HAS domains of the HerA hexamer and the two layers of DUF4297 remain more-or-less symmetric, and no noticeable conformational changes were observed compared to the structure without dsDNA and nucleotides. The rest of the complex undergoes drastic conformational changes to open the pore, allowing DNA entry and binding. Instead of a planar, C6-symmetric organization of the HAS domains, the rest of HerA progressively rotate and translate with respect to one another to assemble into a spiral staircase, with subunit F connecting the lowest subunit A and the highest subunit E of the spiral staircase (Supplementary information, Fig. S6a). The open pore has a size wider than the diameter of dsDNA, leading to the engagement of the DNA substrate in the central pore with only four out of the six HerA subunits (subunits B–E in state 1; subunits A–D in state 2). The four DNA-engaged subunits bind the DNA mainly through positively charged residues. Both strands of the DNA substrate interact with the engaged HerA subunits, with most interactions involving the DNA backbone, suggesting a nonspecific mode of interaction that is compatible with sequence-independent DNA translocation (Supplementary information, Fig. S6c). Notably, two basic residues, R284 and K287, from the insertion subdomain interact with the negatively charged DNA backbone. Four pairs of basic residues from the DNA-engaged subunits adopt a spiral staircase organization that is nicely positioned in a helical path to follow the DNA helix precisely in the 5’ to 3’ direction (Fig. 4b). In addition, the DNA-engaged basic residues form a repeated binding pattern along one strand of the DNA to coordinate two nucleotides per subunit, suggesting two-nucleotides translocation of dsDNA at each step which is catalyzed by HerA.

### Nucleotide binding status in the HerA hexamer

Strong densities attributed to adenine nucleotides were observed at the interface formed by the RecA-like domains of two adjacent subunits, a position similar to the ATP-binding site of archaeal HerA.^24^ The high-quality cryo-EM maps enabled us to unambiguously define the nucleotide binding status for each subunit within the HerA hexamer (Supplementary information, Figs. S4g, h, S5h). For state 1, subunit F and DNA-engaged subunit E bind the ADP molecule, whereas three other DNA-engaged subunits B–D bind the ATP molecule (Fig. 4e, AMPPNP or ATPγS in the structures). Interestingly, subunit A, which is located at the lowest position of the spiral staircase, can bind either ATP (ATPγS in the structure) or ADP molecule, suggesting that this site may be the nucleotide exchange or ATP loading position. For state 2, three DNA-engaged subunits A–C bind ATP (AMPPNP in the structure), and the remaining subunits E–F bind the ADP molecule (Fig. 4e). Strikingly, only weak density in the ATP-binding pocket of DNA-engaged subunit D was observed, suggesting ADP may dissociate at this site in the current structure. The presence of ADP in the ATP-binding pocket can be explained by the low AMPPNP and ATPγS hydrolysis activity of the complex (Supplementary information, Fig. S6f). Residues E426 and E427 from the canonical Walk A motif and S158 of Walk B motif maintain the adenine nucleotides at the active site through magnesium to coordinate the phosphate groups, while K157 of Walk A motif directly contacts with the γ-phosphate of ATP (Fig. 4c). Furthermore, two positively charged residues R455 and K456 from the clockwise adjacent subunit *in trans* interact with the nucleotide phosphate groups of ATP but not ADP (Fig. 4c), which is reminiscent of the critical ‘arginine finger’ or ‘R finger’ of oligomeric ring-shaped ATPase proteins.^27^ Mutating the basic residues of Walk A and R finger drastically reduced the ATPase activity of the DUF4297–HerA complex (Supplementary information, Fig. S10a).

### Translocation mechanism of HerA

The two states resolved here apparently represent two active translocation states on the DNA substrate, enabling us to deduce information about the putative DNA translocation process. We speculated that state 1 will eventually transition into state 2 during the translocation cycle. The presence of ATP in the ATP-binding site is sensed by the R finger from the adjacent subunit, which makes close contact with the γ-phosphate of ATP, consequently pulling the clockwise adjacent subunit up and then strengthening inter-subunit interaction (Supplementary information, Fig. S6b, d). ATP hydrolysis causing loss of the γ-phosphate releases the R finger, repositioning the adjacent subunit. Of note, the R finger of the nucleotide exchange site (subunit A in state 1) lies in a position midway between the ATP and ADP states (Supplementary information, Fig. S6b). Based on the nucleotide binding status and DNA binding observed in these two states, we deduced that ATP hydrolysis in the DNA-engaged subunit D in state 1 would abolish the interaction of the γ-phosphate with the *trans*-acting R finger residues from the clockwise adjacent subunit E, thus weakening the subunit–subunit interaction. This drives a major downward movement of the domains from subunit E that displaces the DNA-interacting residues, thus dissociating with DNA. Meanwhile, nucleotide exchange that occurs in the lowest DNA-disengaged subunit A is sensed by the R finger of subunit B, driving an upward movement of subunit B together with subunits C–D and DNA substrate (Fig. 4d). Consequently, DNA moves upwards, enabling the lowest subunit A to bind the successive two nucleotides at the 5’ side of the DNA substrate.

### DUF4297–HerA assembly promotes dimerization of the DUF4297 domain of the top layer DUF4297

**Fig. 5:**
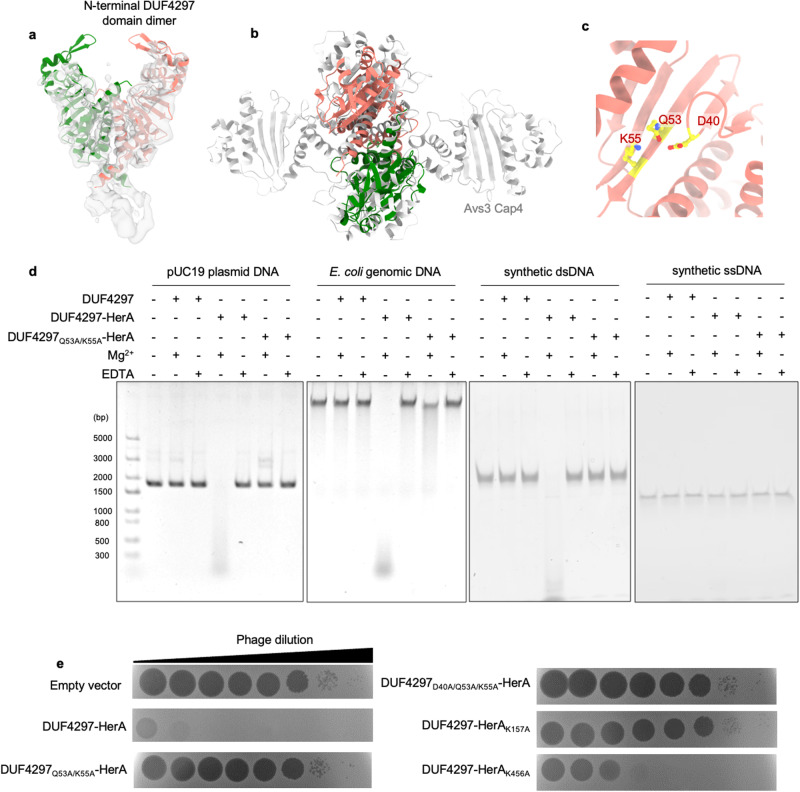
Dimerization of the N-terminal DUF4297 domain. **a** Orthogonal views of the predicted N-terminal domain fitted into the 4.2 Å local-refined cryo-EM map. **b** Superimposition of the N-terminal DUF4297 domain dimer and the Avs3 cap4 domain tetramer. **c** Active site of the N-terminal DUF4297 domain. **d** Agarose gel analysis of the nuclease activity of the DUF4297–HerA complex in vitro with a pUC19 plasmid DNA, *E. coli* genomic DNA, synthetic dsDNA, or synthetic ssDNA. **e** Plaques of phage λ on cells expressing empty vector, DUF4297–HerA WT, and the indicated mutants. 10-fold serial dilutions of the phage lysate were dropped on the plates.

The densities of the DUF4297 domains of the upper layer DUF4297 molecules are apparent in all cryo-EM datasets. Specifically, in the dataset of the sample with ATPγS and DNA, non-alignment 3D classification revealed two distinct classes with almost equal particle populations, with three densities adopting more-or-less C3 symmetry. Superimposition of these two classes showed that the linkers connecting the N-terminal DUF4297 domain and the CTD display different conformations (Supplementary information, Fig. S7a, b). Further particle symmetry expansion and focused local refinement yielded two maps with an overall resolution of ~4.2 Å. The two maps align well, indicating that the DUF4297 domains in these two classes adopt a similar conformation (Supplementary information, Fig. S7c). Two AlphaFold2-predicted DUF4297 domains fit nicely into the density, revealing dimerization of the N-terminal DUF4297 domain of the upper layer DUF4297 proteins (Fig. 5a). The dimer structure is clamp-shaped with αA and two C-terminal helices αE and αF from each domain forming the major interface with a buried surface area of 2028 Å^2^ (Fig. 5a; Supplementary information, Fig. S7d, e). We used the Dali server to search the DUF4297 dimer against entries in the Protein Data Bank, which identified nuclease domains of *Se*Avs3 of NLRs (PDB ID: 8DGC) and *Ec*Cap4 of CBASS (PDB ID: 7YIB) as the two top hits with Z-scores of 14.1 and 12.0, and RMSD of 4.2 Å and 3.1 Å, respectively, despite the fact that they share only respective 18.2% and 23.4% sequence identity with the DUF4297 domain. Like DUF4297 domains, the endonuclease domains of *Se*Avs3 and *Ec*Cap4 are also members of the PD-(D/E)xK-family nuclease. Binding of viral proteins to the sensor domain of *Se*Avs3 triggers *Se*Avs3 tetramerization, which brings adjacent N-terminal nuclease domains close together, forming two nuclease dimers and activating the DNA nuclease activity to abrogate phage infection.^13^ Only the dimer formed by two ‘inward-facing’ subunits possesses DNA cleavage activity.^13^ Structural superimposition revealed that the N-terminal DUF4297 domain dimer adopts a similar configuration as that of ‘inward-facing’ subunits, promoting us to examine whether DUF4297 is an active nuclease (Fig. 5b, c).

### DUF4297–HerA is a dsDNA-targeting nuclease

Given the similar overall structure of the N-terminal DUF4297 domain dimer as that of active endonuclease domains of *Se*Avs3 and its ability to defend against phage infection, we analyzed the in vitro nuclease activity of the DUF4297–HerA complex. Incubation of dsDNA in the presence of DUF4297–HerA complex and Mg^2+^ resulted in the accumulation of smear degradation products, suggesting that DUF4297–HerA is a dsDNA nuclease (Fig. 5d). This nuclease activity depended on the predicted catalytic residues of DUF4297, as mutating the residues of the conserved PD-(D/E)xK motif significantly decreased the nuclease activity (Fig. 5d; Supplementary information, Fig. S8g). Further investigation of the substrate specificity of the complex showed that the nuclease activity is specific for dsDNA. Upon incubating DUF4297–HerA with a ssDNA, ssRNA, or dsRNA substrate, no cleavage product was observed (Fig. 5d; Supplementary information, Fig. S8c). Moreover, the complex cleaved both linear and circular dsDNA, including *E. coli* genomic DNA, indicative of nuclease activity with no specificity for phage DNA. Interestingly, the presence of ATP slightly reduced the nuclease activity, presumably due to negatively charged ATP affecting the binding between dsDNA and DUF4297 (Supplementary information, Fig. S8a). We also found that DUF4297 alone can cleave dsDNA; however, the activity is ~300-fold lower than that of the complex (Supplementary information, Fig. S8e). Mutations disrupting the hydrophobic core of the N-terminal DUF4297 dimer significantly decreased the yield of the DUF4297–HerA complex and drastically reduced the nuclease activity, suggesting that dimerization of the DUF4297 domain of the top layer DUF4297 is crucial for both the complex formation and efficient DNA cleavage (Supplementary information, Fig. S9). Taken together, these experimental data indicate that the DUF4297–HerA complex is an efficient promiscuous DNA nuclease dependent on the dimerization of the DUF4297 domain.

### The DUF4297–HerA dual enzyme complex defends against phage via abortive infection

**Fig. 6:**
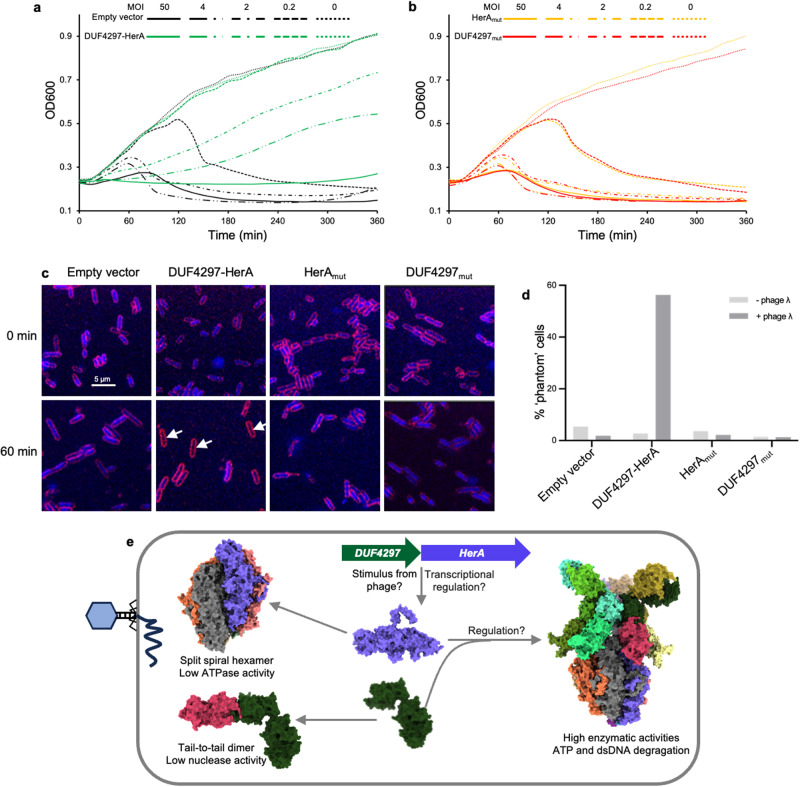
Mechanism of anti-phage defense by DUF4297–HerA. **a** Bacterial growth curves in the presence of DUF4297–HerA upon infection by phage λ with the indicated MOI values. **b** Bacterial growth curves in the presence of DUF4297–HerA mutants (DUF4297_mut_: DUF4297_D40A/Q53A/K55A_–HerA; HerA_mut_: DUF4297–HerA_K157A_) upon infection by phage λ with the indicated MOI values. **c** Representative images of cells expressing DUF4297–HerA or mutants either uninfected or 60 min post infection with phage λ at an MOI of 1. Cell membranes were stained with FM4-64 (red), and DNA was stained with DAPI (blue). White arrows indicate the ‘phantom’ cells devoid of both phage and host DNA. **d** Quantification of the percentage of ‘phantom’ cells in **c**. **e** Assembly and possible activation mechanisms of the DUF4297–HerA complex.

Results of plaque assays showed that the DUF4297–HerA system protects *E. coli* against phage λ, reducing the plating efficiency by five orders of magnitude. Mutations disrupting the ATPase or nuclease active sites abolished defense, suggesting that both enzymatic activities are indispensable for DUF4297–HerA defense (Fig. 5e). We next performed liquid culture growth assays to test whether DUF4297–HerA defends against phage infection via growth arrest or suicide of infected cells to prevent virus reproduction. Under low multiplicity of infection (MOI = 0.2–4) conditions, bacteria containing the DUF4297–HerA system survived at slow growth rates, but bacteria without the DUF4297–HerA system died. The DUF4297–HerA system diminished defense at a high MOI (MOI = 50), consistent with an abortive infection phenotype in which infected cells died to prevent viral spread (Fig. 6a). Consistent with the results of plaque assays, bacteria with the DUF4297–HerA system harboring ATPase-defective or nuclease-defective mutation died of phage infection at both low and high MOI (Fig. 6b). We further visualized this system responding to phage infection in vivo using fluorescence microscopy. We observed that upon phage infection, DUF4297–HerA system-containing cells degrade both host DNA and phage DNA, creating ‘phantom’ cells that are devoid of DNA (Fig. 6c, d). 56.3% of cells containing WT system lack visible DNA at 60 min post-infection with phage λ under the testing condition. When the ATPase or nuclease active site was mutated, the accumulation of ‘phantom’ cells disappeared. This is in good agreement with our in vitro biochemical data that DUF4297–HerA efficiently degrades both linear and circular DNA. Collectively, these results suggest that the DUF4297–HerA system protects bacteria from phage infection by degrading both host and phage DNA, and reinforce the notion that both ATPase and nuclease activities are indispensable for DUF4297–HerA defense.

## Discussion

Our results demonstrate that DUF4297 and HerA assemble into a supramolecular complex, forming a dual-enzyme defense system with promiscuous dsDNA nuclease and ATPase activities. DUF4297 alone exists as a tail-to-tail dimer with limited nuclease activity, while HerA alone forms a nonplanar split spiral hexameric assembly independent of ATP and DNA, a conformation unfavorable for ATP hydrolysis and DUF4297–HerA complex formation. The enzymatic activities and anti-phage defense function of the DUF4297–HerA system are activated through supramolecular assembly.

Structural comparison immediately explains why the ATPase activity of HerA alone is inhibited. While the pocket created by Walk A and Walk B motifs can accommodate the ATP molecule, relocation of the R finger is needed to hydrolyze ATP because it is too far away (20 Å) from the Walker A motif with which they form the ATPase active site. However, the required conformational change would cause a steric clash at the insertion and four-helix bundle domains, hindering the required movement of the R finger (Supplementary information, Fig. S6d, e, g). By combining structural analysis with biochemical assays, we show that DUF4297–HerA complex formation is required for high enzymatic activities. Complex formation promotes the dimerization of the N-terminal DUF4297 of the top layer DUF4297, thus activating its DNA nuclease activity. Meanwhile, HerA in the complex adopts a conformation capable of hydrolyzing ATP efficiently, which can be further slightly stimulated by the DNA fragments produced by DUF4297 (Supplementary information, Fig. S10). Our data suggest that the DUF4297–HerA system achieves anti-phage function by degrading host and phage dsDNA and potentially depleting the essential molecule ATP to efficiently eliminate phage infection. Despite ATPase and nuclease activities being simultaneously activated through supramolecular assembly, these two enzymatic activities are mutually independent in vitro. Mutations dismissing one enzymatic activity showed a limited effect on the other enzymatic activity in vitro (Supplementary information, Figs. S8b, S10a). However, it is a totally different scenario in vivo. We did not observe significant accumulation of the ‘phantom’ cells when the ATP-binding site was mutated in the phage infection and protein overexpression experiments (Fig. 6c, d; Supplementary information, Fig. S11b, c), indicating that the ATPase activity is important for the DUF4297 nuclease activity in vivo. The bacterial chromosomal DNA forms a compact structure called nucleoid, which is maintained by many nucleoid-associated proteins (NAPs).^28^ Binding of these NAPs to the bacterial genomic DNA can protect DNA from nuclease degradation.^29^ The size of the continuous pore formed by the HerA hexamer and the top layer DUF4297 is large enough to allow the passage of dsDNA. We modeled a dsDNA into the central pore of the DUF4297–HerA complex. Based on this model, it is possible that the DNA will be translocated towards the top layer DUF4297, fueled by ATP hydrolysis (Supplementary information, Fig. S7f). Consequently, the NAPs bound to the dsDNA will be displaced; and then, the free DNA will be more efficiently grabbed and cut by the activated N-terminal DUF4297 domain dimer. Our biochemical analyses and fluorescence microscopy data demonstrate that the DUF4297–HerA complex is constitutively active in vitro, and the complex is toxic to cells (Supplementary information, Fig. S11), suggesting that the enzymatic activities of this system are tightly negatively regulated and unleashed upon phage infection, or that the DUF4297–HerA complex is only expressed upon phage infection (Fig. 6e). The mechanism by which phage infection triggers the activation of the DUF4297–HerA system awaits further investigation.

Ring-shaped oligomeric ATPase proteins, including members from the AAA+ superfamily and FtsK–HerA superfamily, play essential roles in a variety of biological processes. They can utilize energy from ATP hydrolysis to remodel or translocate along many macromolecules such as nucleic acids and unfolded polypeptides.^27,30^ Extensive studies have revealed that AAA+ proteins usually exist as monomers or form symmetric oligomers without substrates. Recent technological and methodological developments in cryo-EM have enabled the structure determination of a plethora of cryo-EM structures of AAA+ proteins in complex with their translocated substrates, suggesting a universal mechanism that the AAA+ proteins adopt an asymmetric spiral staircase conformation to encircle substrates and facilitate the translocation of the substrate through the central pore.^31–35^ By binding to the backbone of nucleic acids or polypeptide substrates, AAA+ proteins can translocate substrate in a sequence-independent manner. The substrate translocation mechanisms in previous AAA+ proteins-related studies were often proposed based on only one structure in an active translocation state. Two high-resolution cryo-EM structures representing different translocation active states and the structure without substrate engagement that we present here provide key snapshots during DNA translocation cycles and crucial insights into the DNA translocation mechanism of these ring-shaped ATPases. Upon nucleotide and DNA binding, HerA undergoes a large-scale conformational change to form a right-handed spiral staircase that allows four HerA subunits to interact with two nucleotides of one DNA strand per subunit following the DNA’s helix precisely in the 5’ to 3’ direction. ATP hydrolysis and binding drive conformational changes within the spiral staircase through the conserved R finger. ATP hydrolysis and binding cycle proceeds around the hexameric ring in a counterclockwise manner when viewed from the bottom of HerA. This sequential model is similar to the mechanisms proposed for DNA motor proteins Ftsk and RuvB hexamer.^31–33^ During each ATP binding and hydrolysis cycle, the DNA-engaged subunit at the top of the spiral staircase (subunit E in state 1 and subunit D in state 2) disengages from DNA, while counterclockwise adjacent subunit of the DNA-engaged subunit at the bottom of the spiral staircase (subunit A in state 1 and subunit F in state 2) contacts the successive two nucleotides at the 5’ side of the DNA substrate.

In summary, our work elucidates the molecular basis for the function of DUF4297–HerA system, revealing a unique and interesting mechanism for the activation of a dual-enzyme anti-phage defense system facilitated by supramolecular assembly.

## Materials and Methods

### Protein expression and purification

For the expression and purification of the DUF4297–HerA complex, DNA sequences encoding DUF4297 (WP_016239654.1) and HerA (WP_016239655.1) were cloned into a pET28a vector containing a His tag at the N-terminus of DUF4297 and transformed into *E. coli* BL21(DE3) cells. For DUF4297, protein expression was induced by the addition of isopropyl β-_D_-thiogalactopyranoside (IPTG) to a final concentration of 0.4 mM at 16 °C when the OD600 reached 0.6–0.8, and the cells were cultured for another 20 h. Cells were harvested via centrifugation at 4900× *g* for 10 min at 10 °C. The collected cells were then resuspended in lysis buffer (20 mM Tris-HCl, pH 8.0, 150 mM NaCl, and 20 mM imidazole) and lysed using a high-pressure cell crusher (Union-Biotech) at 600 bars. The resulting lysate was centrifuged at 30,000× *g* for 60 min at 4 °C. The supernatant was collected, loaded onto Ni-charged Resin FF (GenScript), and washed with 50 mL of wash buffer (20 mM Tris-HCl, pH 8.0, 150 mM NaCl, and 60 mM imidazole). The target protein was eluted with the elution buffer containing 20 mM Tris-HCl, pH 8.0, 150 mM NaCl, and 500 mM imidazole. Subsequent purification was performed using a Superose 6 Increase 10/300 GL size exclusion chromatography column (Cytiva) pre-equilibrated with buffer containing 20 mM Tris-HCl, pH 8.0, and 150 mM NaCl. Peak fractions containing the DUF4297–HerA complex were pooled and concentrated to ~5 mg/mL and stored at −80 °C for future use. Expression and purification of HerA, DUF4297–HerA complex, and mutants followed a similar procedure.

### Cryo-EM sample preparation

For the preparation of cryo-EM samples of HerA and DUF4297–HerA complex, 3.5 μL of purified proteins at a concentration of ~1.0 mg/mL were applied to the glow-discharged Cu 200 mesh R1.2/1.3 holey carbon grids (Quantifoil). After a 20-s incubation, the grids were blotted for 2 s with a blot force of 0 at 100% humidity and 4 °C, then plunge-frozen using Vitrobot Mark IV (FEI, Thermo Fisher Scientific). For the DUF4297–HerA–AMPPNP–dsDNA sample, 3.5 μL of the purified sample with 1 mM AMPPNP and 5 mM MgCl_2_ at a concentration of ~1.0 mg/mL was incubated with a 59-bp dsDNA at a molar ratio of 1:1.2 for 30 min on ice. The mixture was then subjected to the same plunge-freezing procedure. For the preparation of the DUF4297–HerA–ATPγS–dsDNA complex, the purified complex carrying mutations (Q53A/K55A on DUF4297) with 2 mM ATPγS and 5 mM MgCl_2_ was incubated with a 59-bp dsDNA at a molar ratio of 1:1.4 for 30 min on ice. The assembled complex was then employed for cryo-EM sample preparation, following a similar protocol used for the DUF4297–HerA complex.

### Cryo-EM data collection and image processing

Cryo-EM data were collected with a CRYO ARM 300 electron microscope (JEOL, Japan) operating at 300 kV, with a K3 direct electron detector (Gatan, United States). Data were collected at a nominal magnification of 50,000× in super-resolution counting mode, with a super-resolution pixel size of 0.475 Å/pixel. Movies were automatically collected using Serial-EM software^36^ at a frame rate of 40 frames per second, accumulating a total dose of 40 e/Å^2^ within a defocus range of −0.5 to −2.5 μm. Patch-based motion correction and CTF estimation of the recorded movies were performed in cryoSPARC v4.2.0.^37^ Following this correction and estimation, low-quality images were excluded.

For HerA, particles were automatically picked by Blob picker from 3034 micrographs, extracted with a particle box size of 240 pixels, and subsequently subjected to 2D classification. 756,562 particles from good classes were selected for further heterogeneous refinement requesting six classes. The most optimal class containing 272,576 particles was selected for subsequent 3D classification. One class forming intact hexamer was selected for further NU-refinement, yielding a map with an overall resolution of 3.14 Å.

For the DUF4297–HerA complex, particle picking was performed using Topaz picking from 4657 micrographs. Subsequently, 548,250 particles from good 2D classes were subjected to heterogeneous refinement, requesting four classes. One class containing 339,543 particles was then selected for 3D classification. Two of the 3D classification classes containing 290,611 particles were selected and subjected to NU-refinement with C2 symmetry imposed, resulting in a 2.87 Å reconstruction.

For the DUF4297–HerA–AMPPNP–dsDNA complex, 1,375,531 particles picked by Topaz picking from 6042 micrographs were subjected to 2D classification, followed by further heterogeneous refinement and 3D classification, requesting four classes, respectively. Two distinct classes, including intact protein complex and DNA, were identified from 3D classification and selected for further NU-refinement, resulting in two final reconstructions with overall resolutions of 2.73 Å and 2.92 Å, respectively.

For the DUF4297–HerA–ATPγS–dsDNA complex, 740,397 particles were picked from 3765 micrographs using Topaz picking. After subsequent 2D classification and heterogeneous refinement, one class containing 290,376 particles was selected for further 3D classification, requesting four classes. Two classes (class 1 and class 2) display strong densities of the N-terminal DUF4297 domain of the top layer DUF4297 with almost equal particle populations were selected for further NU-refinement, yielding a consensus 2.81 Å map. The two classes were also selected for particle expansion with C3 symmetry, focused 3D classification, and then focused local refinement, yielding 4.22 Å and 4.34 Å reconstructions, respectively.

### Model building and refinement

DUF4297 and HerA model predicted by AlphaFold2 were used as the initial models.^38^ These predicted models were fitted into the cryo-EM maps using Chimera. The initial models were then manually inspected and rebuilt in Coot.^39^ The models were refined in Phenix using phenix.real_space_refine.^40^ The nucleotides and DNA were manually built to fit the densities, followed by iteratively refined with phenix.real_space_refine. The quality of the final models was analyzed with MolProbity in Phenix.^41^ Refinement statistics are summarized in Supplementary information, Table S1.

### Analytical ultracentrifugation

The sedimentation velocity measurement was carried out using a Beckman Optima Analytical Ultracentrifuge (Beckman Coulter) with an An-60 Ti rotor at 55,000 rpm at 4 °C. Purified proteins were diluted to ~1 mg/mL in 390 μL TN buffer (20 mM Tris-HCl, pH 8.0 and 500 mM NaCl). TN buffer was used as a reference at a volume of 400 μL. Absorbance at 280 nm was measured every 60 s. The sedimentation coefficient was analyzed by SEDFIT and SEDPHAT programs.^42^

### In vitro nuclease activity assays

Purified proteins were incubated with nucleic acid substrates in a reaction buffer containing 20 mM HEPES, pH 7.5, 50 mM NaCl, 1 mM DTT, 5% glycerol. A total of 10 μL reaction contains 2 nM DUF4297–HerA complex or mutants (DUF4297 alone was proportionally added to a final concentration of 24 nM), 200 ng pUC19 plasmid DNA or *E. coli* genomic DNA. 5 mM MgCl_2_ or EDTA was added in the corresponding reaction. The reaction was carried out at 37 °C for 30 min. The reaction was quenched by the addition of 4 μL stop buffer containing 150 mM EDTA, pH 8.0, 1.5% SDS, and 20% glycerol and then loaded onto a 1% agarose gel. Gels were run for 35 min at 130 V, stained with GoldView nucleic acid stain, and then imaged with a ChemiDoc Imaging System (Biorad). For synthetic DNA cleavage assays, 10 nM DUF4297–HerA complex or 120 nM DUF4297 was incubated with 1 μM dsDNA or 2 μM ssDNA. The products were analyzed by 10% native PAGE.

### In vitro ATPase activity assays

The ATPase activity of the DUF4297–HerA complex and mutants was measured using a modified Baginski method.^43^ Briefly, the 50 μL reaction volume contains 5 nM DUF4297–HerA or mutants (HerA alone was added to a final concentration of 200 nM to obtain an accurate absorbance value), 20 mM Tris-HCl, pH 8.0, 50 mM NaCl, 5 mM MgCl_2_, and 5 mM DTT. Indicated concentration of ATP was added to start the reaction, and the mixture was incubated at 37 °C for 30 min. The reaction was stopped by adding 100 μL of freshly prepared Solution II (2.86% ascorbic acid, 1 M HCl, 0.48% (NH_4_)_2_MoO_4_, 2.86% SDS) and incubated on ice for 10 min. Then, 150 μL Solution III (3.5% Bismuth citrate, 1 M HCl, and 3.5% sodium citrate) was added and incubated on ice for another 10 min. Absorbance was measured at 710 nm. Meanwhile, a standard curve was used to determine the release of sample Pi using K_2_HPO_4_.

### Phage cultivation and plaque assays

Phage λ was lined in the double-layer agar plate and incubated overnight with host bacteria *E. coli* K-12 MG1655 at 37 °C. Plaques were scraped in 2 mL Luria Broth (LB), and the supernatant was filtered through with a 0.22-μm sterile filter. Phage titer was determined using the small drop plaque assay method.^44^ *E. coli* BL21(DE3) host cells containing an empty vector with native promoter sequence of the *DUF4297–HerA* operon, the DUF4297–HerA system or mutants, were grown overnight at 37 °C in LB. 3 mL *E. coli* culture was added into 10 mL top agar (0.75% agar), and the mixture was poured on 10-cm LB-agar plates containing 25 μg/mL kanamycin and incubated for 1 h at 37 °C. The phage containing 6 × 10^9^ virions was serially diluted 10-fold with LB, and 2.5 μL of diluted phages was spotted onto the double-layer agar. The plates were recorded by BIO-RAD GelDoc Go after overnight incubation at 37 °C.

### Liquid culture growth assays

Early-log culture (180 μL per well) was transferred into a 96-well plate containing phage lysate (20 μL per well) for infection at an indicated MOI. Infections were performed in triplicates with overnight cultures prepared from three separate colonies. Plates were incubated at 37 °C with continuous shaking in BioTek Synergy H1 Plate Reader. The OD600 values were measured every 15 min for 6 h.

### Negative stain EM and reconstruction

Purified DUF4297 protein in buffer containing 20 mM Tris-HCl, pH 8.0, and 500 mM NaCl was added onto carbon film-coated 300 mesh Copper grids at a concentration of ~100 nM, and stained with 2% uranyl acetate. Grids were imaged on a Talos L120C (Thermo Fisher Scientific) electron microscope with a CETA 16 M detector at 3 Å/pixel and 60 e/Å^2^. The collected micrographs were processed in cryoSPARC v4.2.0, and particles were picked using Blob Picker and extracted with a particle box size of 120 pixels, then subjected to 2D classification and 3D reconstruction.

### Live single-cell static fluorescence microscopy

*E. coli* K-12 MG1655 host cells containing an empty vector with the native promoter sequence of the *DUF4297–HerA* operon, the DUF4297–HerA system or mutants, were grown to OD600 0.3 in LB at 37 °C. Bacterial samples collected at 0 and 60 min after phage infection at MOI of 1 were centrifuged at 3000× *g*. The pellets were resuspended in 50 μL PBS and stained successively with DAPI (final concentration of 7 μM, 30 min) and FM4-64 (final concentration of 32 μM, 30 min) at room temperature. One microliter of the stained bacteria was dropped onto 0.5% solid agarose for visualization by Dragonfly 200 fluorescence microscopy. Image analysis was performed using Imaris Viewer (v10.1.0).

### Supplementary information

Supplementary information, Fig. S1

Supplementary information, Fig. S2

Supplementary information, Fig. S3

Supplementary information, Fig. S4

Supplementary information, Fig. S5

Supplementary information, Fig. S6

Supplementary information, Fig. S7

Supplementary information, Fig. S8

Supplementary information, Fig. S9

Supplementary information, Fig. S10

Supplementary information, Fig. S11

Supplementary information, Table S1

## Data Availability

The cryo-EM maps and atomic coordinates have been deposited to the Electron Microscopy Data Bank (EMDB) and the Protein Data Bank (PDB), respectively, with accession codes: EMD-38203, 8XAU for HerA; EMD-38204, 8XAV for DUF4297–HerA; EMD-38205, 8XAW for DUF4297–HerA–AMPPNP–DNA state 1; EMD-38206, 8XAX for DUF4297–HerA–AMPPNP–DNA state 2; EMD-38207, 8XAY for DUF4297_Q53A/K55A_–HerA–ATPγS–DNA.
