# Supplementary material for: Molecular and structural basis of an ATPase-nuclease dual-enzyme anti-phage defense complex
Source: Cell Res. 2024 Jun 4;34(8):545–55. doi: 10.1038/s41422-024-00981-w (PMC11291478; doi:10.1038/s41422-024-00981-w)
Supplement: Supplementary file 1 — Supplementary information, Fig. S1 [file 41422_2024_981_MOESM1_ESM.pdf]

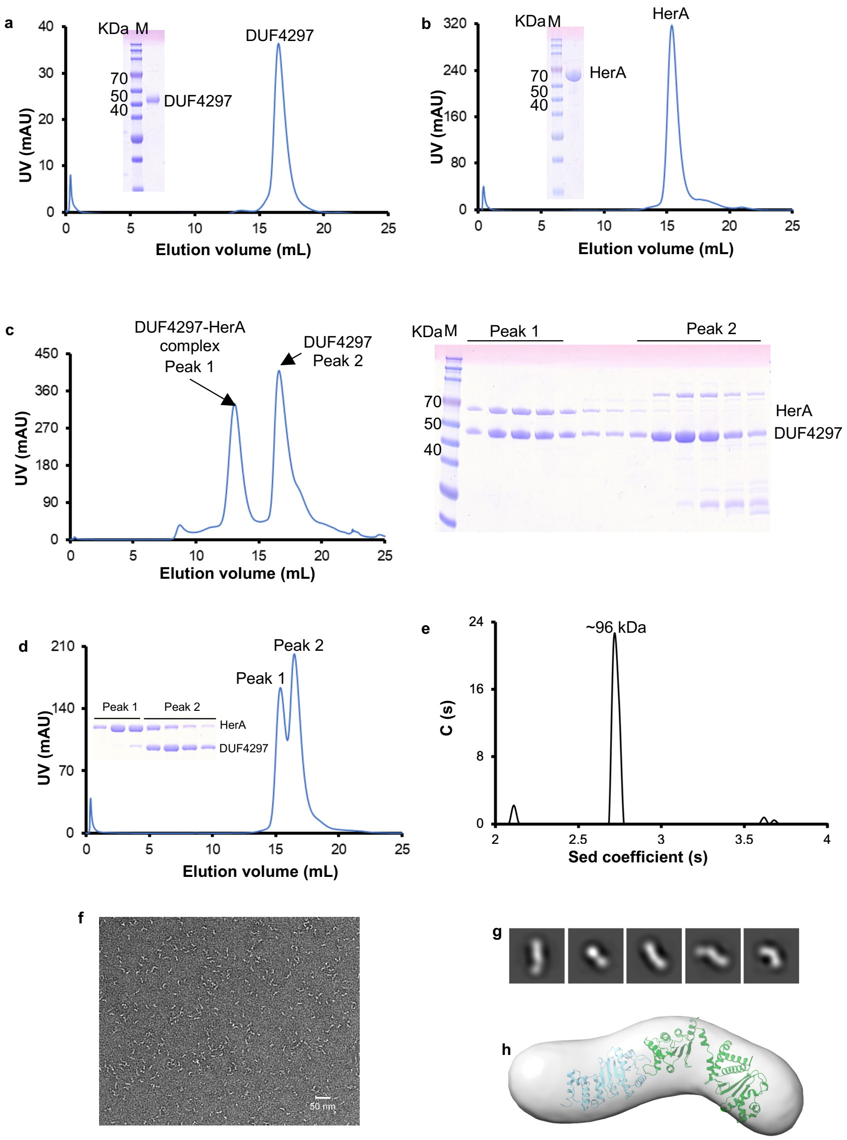

Supplementary information Figure S1. Protein expression and purification. Size-exclusion (Superose 6 Increase 10/300 GL Cytiva) and SDS-PAGE profiles of the purified recombinant proteins: DUF4297 (**a**), HerA (**b**) and DUF4297-HerA complex (**c**). **d** *In vitro* DUF4297-HerA complex reconstitution test. **e** Sedimentation coefficient distribution of DUF4297 alone. DUF4297 alone has a measured mass of ~96 kDa, the theoretical molecular mass of monomer is ~48 kDa. **f** Representative micrograph of negative-stained DUF4297 alone. **g** Reference-free two-dimensional class averages of particles in (**f**). **h** The tail-to-tail DUF4297 dimer fitted into the low-resolution negative stain reconstruction.
