# Supplementary material for: Molecular and structural basis of an ATPase-nuclease dual-enzyme anti-phage defense complex
Source: Cell Res. 2024 Jun 4;34(8):545–55. doi: 10.1038/s41422-024-00981-w (PMC11291478; doi:10.1038/s41422-024-00981-w)
Supplement: Supplementary file 2 — Supplementary information, Fig. S2 [file 41422_2024_981_MOESM2_ESM.pdf]

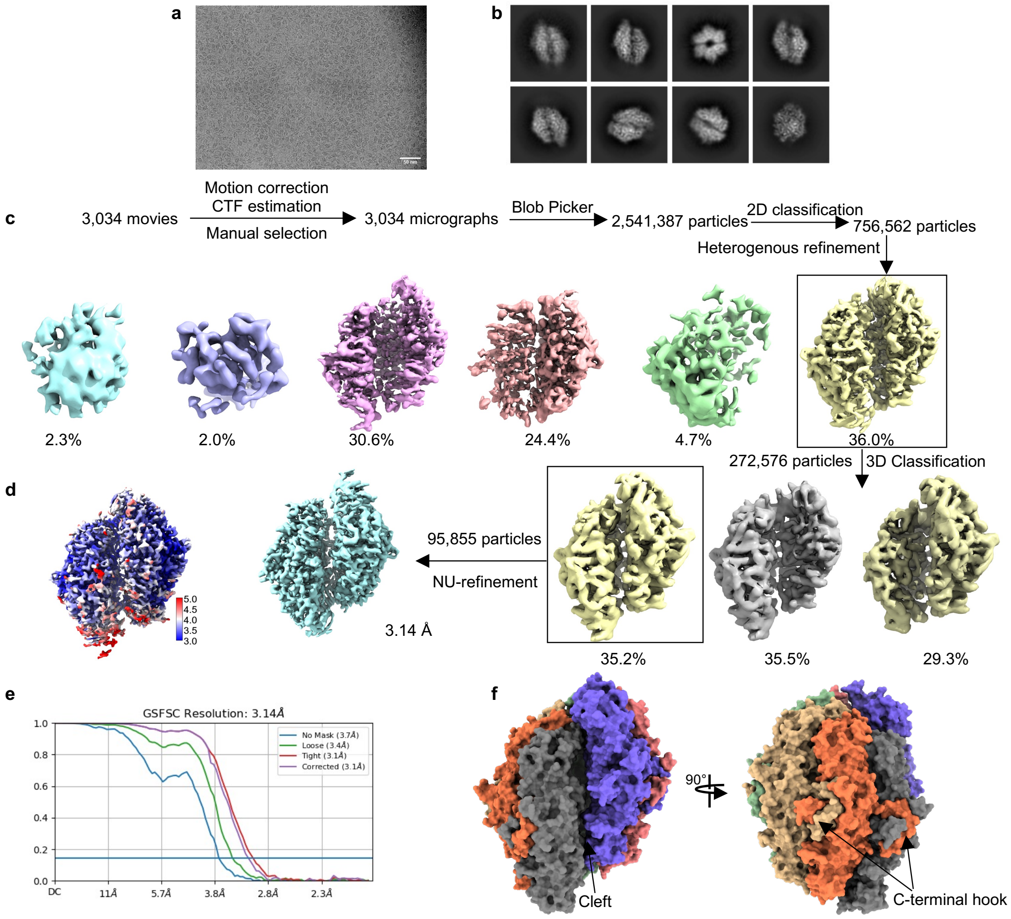

Supplementary information Figure S2. Cryo-EM reconstruction of HerA alone. a Representative cryo-EM image of HerA alone. **b** 2D class averages of HerA alone. **c** Flowchart of cryo-EM data processing. **d** Cryo-EM density map colored by local resolution. **e** Fourier shell correlation (FSC) curve was calculated using two independent half maps, and resolution was estimated using the FSC=0.143 cutoff. **f** Surface representation of nonplanar split spiral HerA is shown to illustrate the cleft and interaction between the C-terminal hook and adjacent subunit.
