# Supplementary material for: Molecular and structural basis of an ATPase-nuclease dual-enzyme anti-phage defense complex
Source: Cell Res. 2024 Jun 4;34(8):545–55. doi: 10.1038/s41422-024-00981-w (PMC11291478; doi:10.1038/s41422-024-00981-w)
Supplement: Supplementary file 3 — Supplementary information, Fig. S3 [file 41422_2024_981_MOESM3_ESM.pdf]

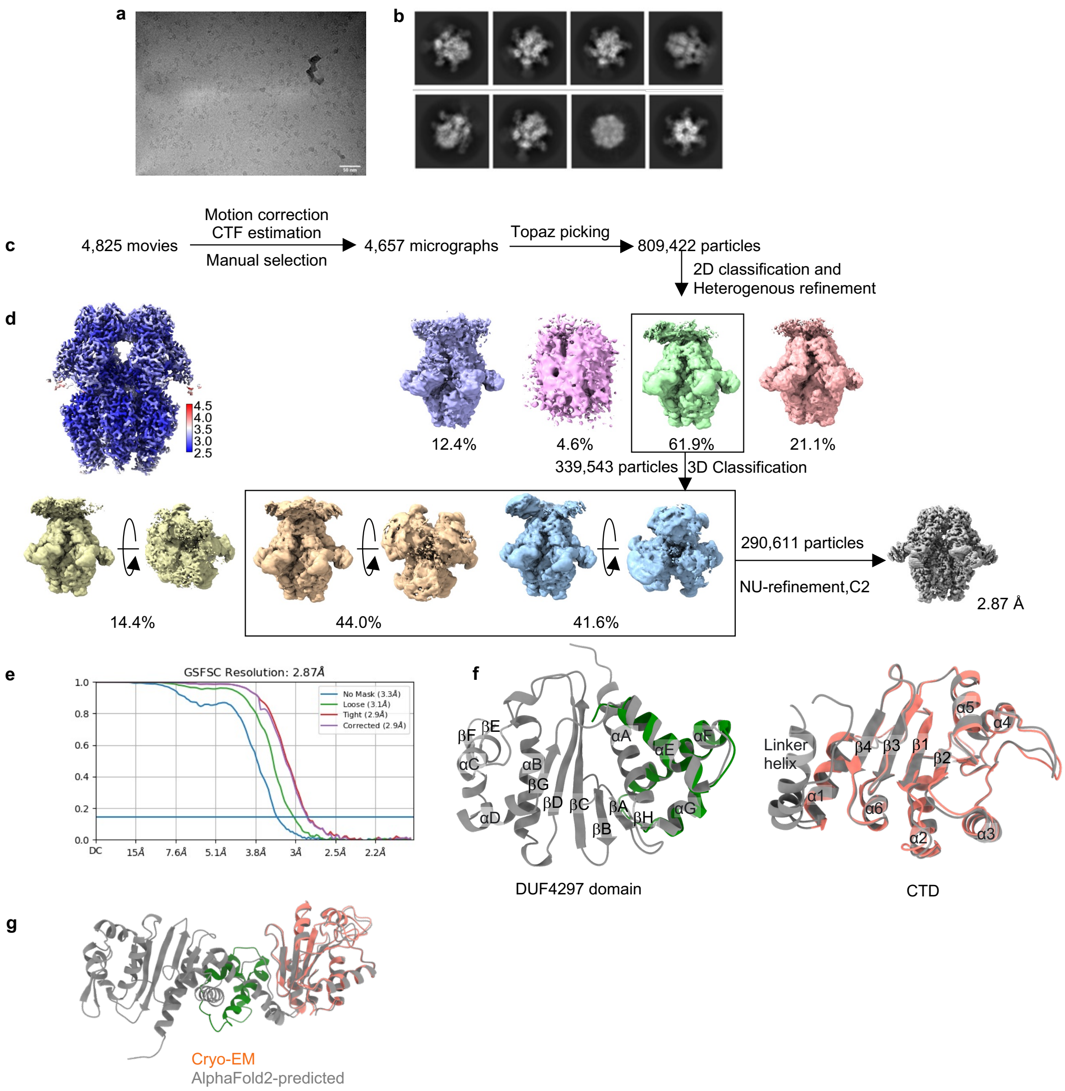

Supplementary information Figure S3. Cryo-EM reconstruction of the DUF4297-HerA complex. **a** Representative cryo-EM image of the DUF4297-HerA complex. **b** 2D class averages of the DUF4297-HerA complex. **c** Flowchart of cryo-EM data processing. **d** Cryo-EM density map colored by local resolution. **e** Fourier shell correlation (FSC) curve was calculated using two independent half maps (left panel), and resolution was estimated using the FSC=0.143 cutoff. Euler angle distribution of the refined particles (right panel). **f** Secondary structures of the N-terminal domain and CTD. Predicted DUF4297 is colored grey. **g** Superimposition of cryo-EM resolved DUF4297 and predicted DUF4297 structures.
