# Supplementary material for: Molecular and structural basis of an ATPase-nuclease dual-enzyme anti-phage defense complex
Source: Cell Res. 2024 Jun 4;34(8):545–55. doi: 10.1038/s41422-024-00981-w (PMC11291478; doi:10.1038/s41422-024-00981-w)
Supplement: Supplementary file 4 — Supplementary information, Fig. S4 [file 41422_2024_981_MOESM4_ESM.pdf]

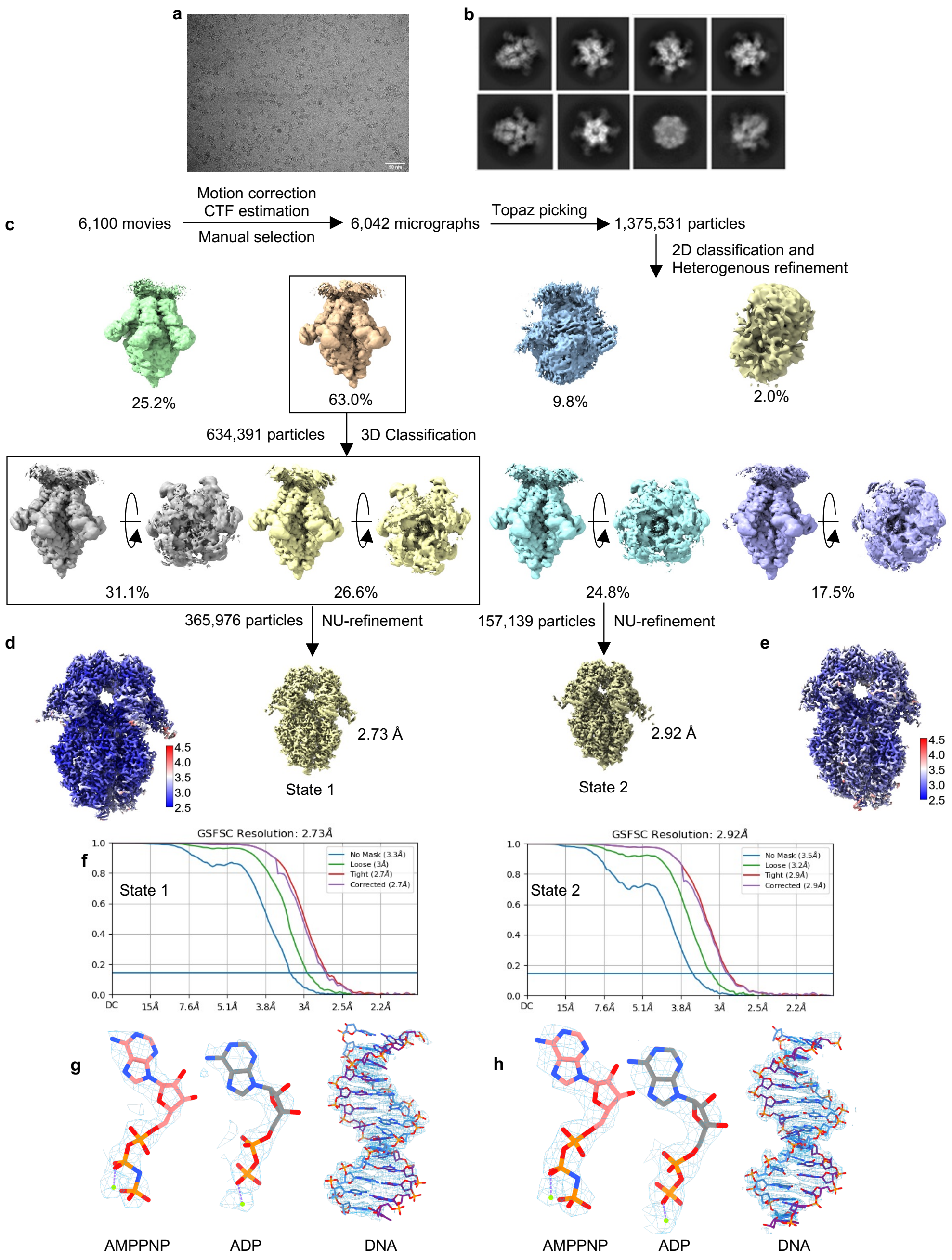

Supplementary information Figure S4. Cryo-EM reconstruction of the DUF4297-HerA complex in the presence of dsDNA and AMPPNP. a Representative cryo-EM image of the DUF4297-HerA complex in the presence of dsDNA and AMPPNP. **b** 2D class averages of the DUF4297-HerA complex in the presence of dsDNA and AMPPNP. **c** Flowchart of cryo-EM data processing. **d-e** Cryo-EM density maps of state 1 and state 2 colored by local resolution. **f** Fourier shell correlation (FSC) curves were calculated using two independent half maps, and resolution

was estimated using the FSC=0.143 cutoff. **g-h** Representative cryo-EM densities of nucleotides and DNA in state 1 (**g**) and state 2 (**h**), respectively.
