# Supplementary material for: Molecular and structural basis of an ATPase-nuclease dual-enzyme anti-phage defense complex
Source: Cell Res. 2024 Jun 4;34(8):545–55. doi: 10.1038/s41422-024-00981-w (PMC11291478; doi:10.1038/s41422-024-00981-w)
Supplement: Supplementary file 5 — Supplementary information, Fig. S5 [file 41422_2024_981_MOESM5_ESM.pdf]

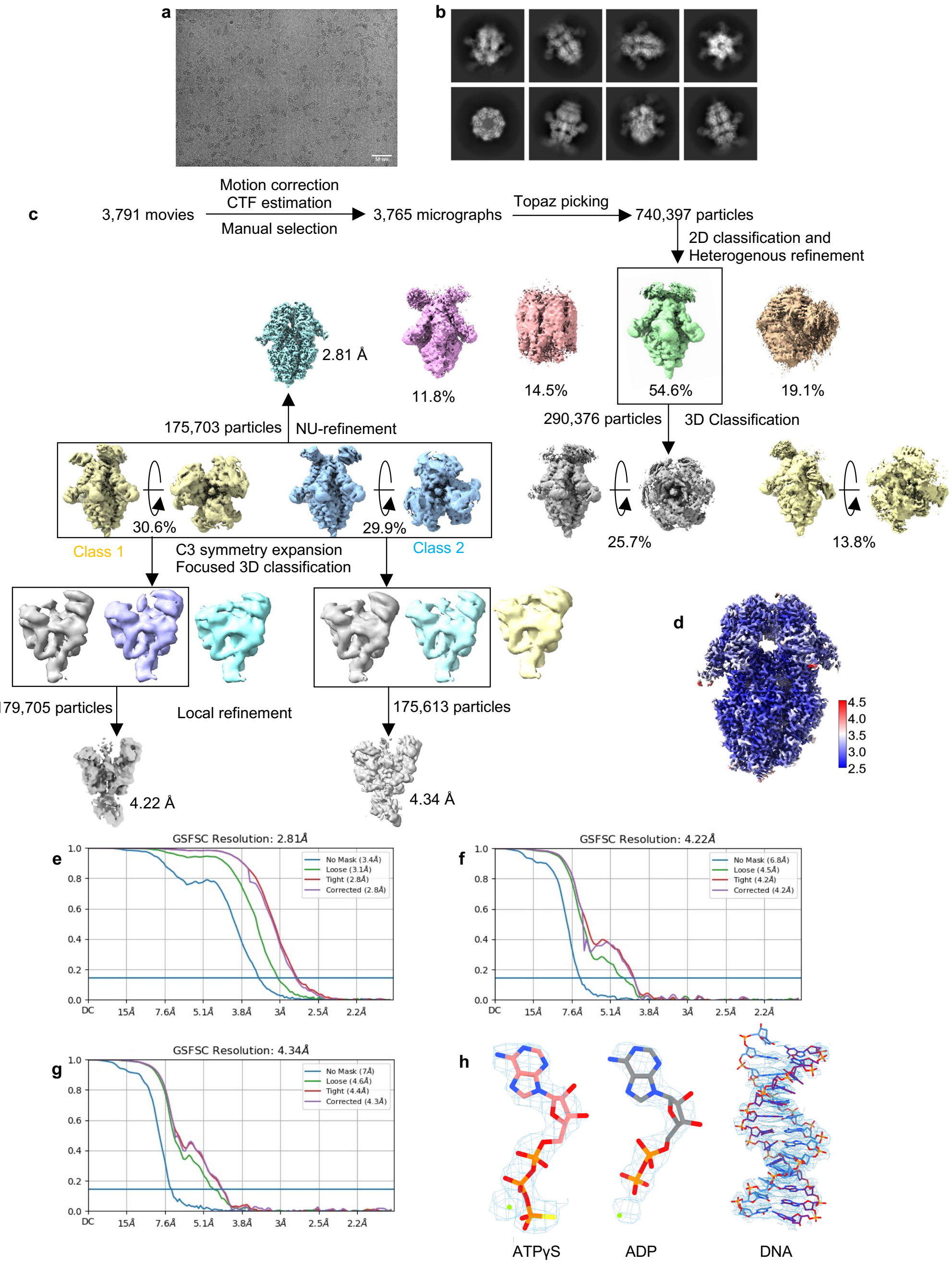

Supplementary information Figure S5. Cryo-EM reconstruction of the DUF4297-HerA complex in the presence of dsDNA and ATPγS. a Representative cryo-EM image of the DUF4297-HerA complex in the presence of dsDNA and ATPγS. **b** 2D class averages of the DUF4297-HerA complex in the presence of dsDNA and ATPγS. **c** Flowchart of cryo-EM data processing. **d** Consensus cryo-EM density map colored by local resolution. **e-g** Fourier shell correlation (FSC) curves of consensus reconstruction (**e**), class 1 (**f**), and class 2 (**g**) were calculated using two independent half maps. **h** Representative cryo-EM densities of nucleotides and DNA.
