# Supplementary material for: Molecular and structural basis of an ATPase-nuclease dual-enzyme anti-phage defense complex
Source: Cell Res. 2024 Jun 4;34(8):545–55. doi: 10.1038/s41422-024-00981-w (PMC11291478; doi:10.1038/s41422-024-00981-w)
Supplement: Supplementary file 6 — Supplementary information, Fig. S6 [file 41422_2024_981_MOESM6_ESM.pdf]

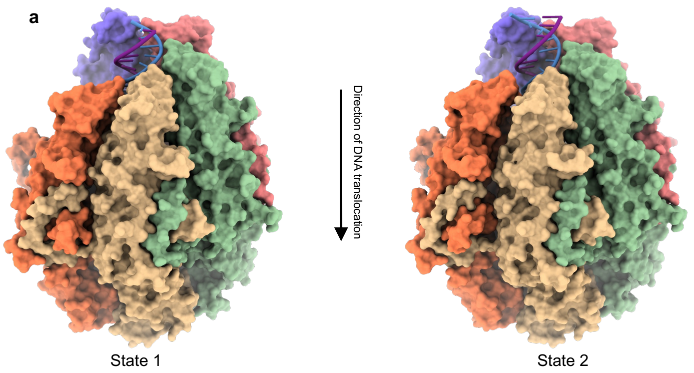

b Distance between WB motif and R finger (Å), distance between Cα of K157 and R455 of clockwise adjacent subunit

State \ Interface	A-B	B-C	C-D	D-E	E-F	F-A
State 1-AMPPNP	16.3	13.6	15.2	15	19.4	19.5
State 1-ATPγS	16.4	14.4	14.4	15.1	19.4	19.8
State 2-AMPPNP	15.4	13.7	15.3	17.2	20	19.5

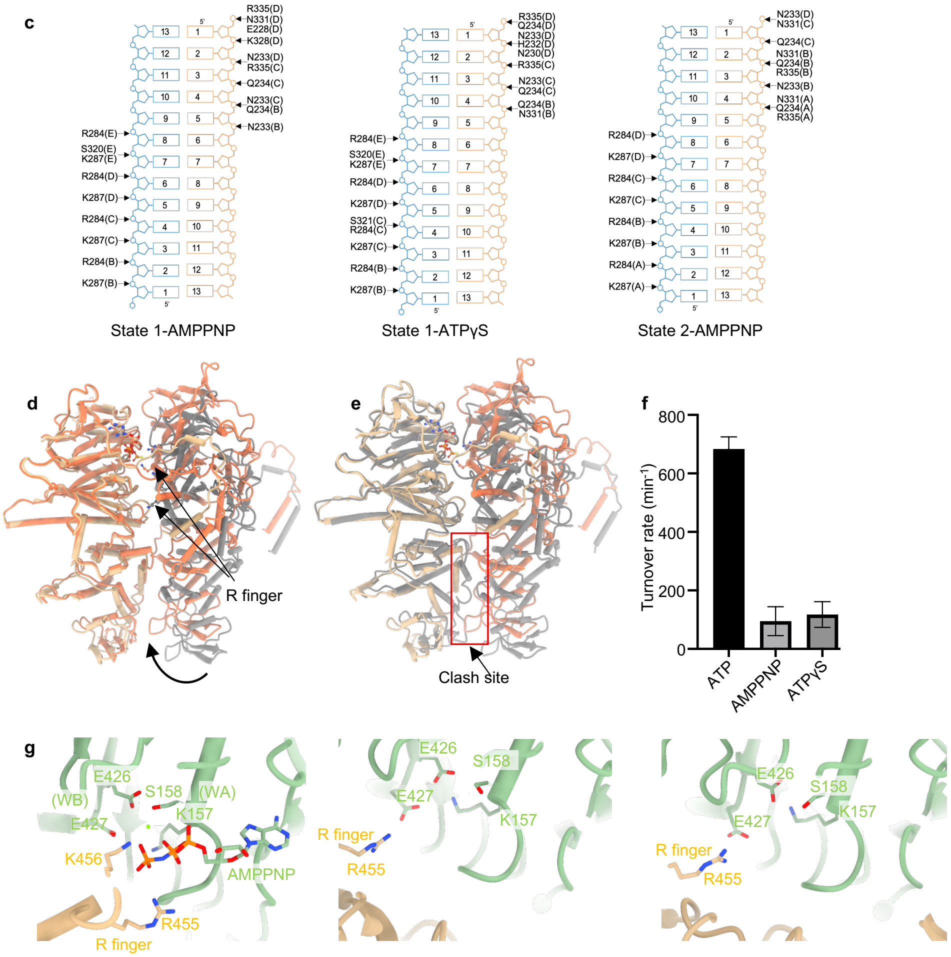

Supplementary information Figure S6. Conformational changes induced by nucleotides and DNA binding. a Surface representation of HerA subunit A-E shows the architecture of a spiral

staircase. **b** Distance between WB motif and R finger in different states. **c** Detailed interactions between HerA and dsDNA. **d** Structural comparison of two adjacent HerA subunits bound to AMPPNP and two adjacent HerA subunits bound to ADP. Subunits binding AMPPNP are colored yellow and orange. Subunits binding ADP are colored orange and grey. **e** Structural comparison of two adjacent HerA subunits bound to AMPPNP and two adjacent HerA subunits of HerA alone hexamer. Subunits of HerA alone are colored grey. **f** Hydrolysis of ATP, AMPPNP or ATPγS by the DUF4297-HerA complex. **g** Conformational details of the ATP binding pocket of DUF4297-HerA-AMPPNP-DNA (left), DUF4297-HerA (middle), and HerA alone (right).
