# Supplementary material for: Molecular and structural basis of an ATPase-nuclease dual-enzyme anti-phage defense complex
Source: Cell Res. 2024 Jun 4;34(8):545–55. doi: 10.1038/s41422-024-00981-w (PMC11291478; doi:10.1038/s41422-024-00981-w)
Supplement: Supplementary file 7 — Supplementary information, Fig. S7 [file 41422_2024_981_MOESM7_ESM.pdf]

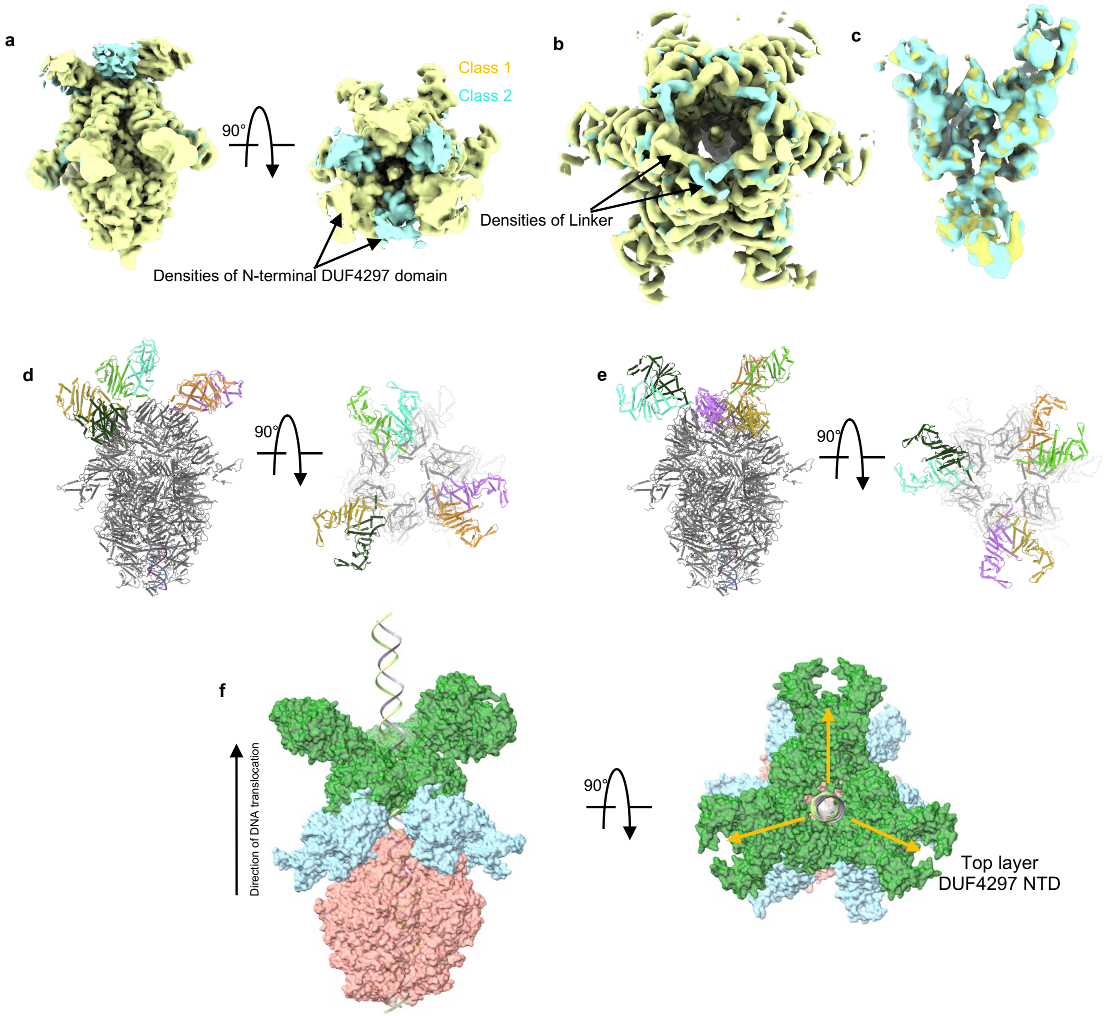

Supplementary information Figure S7. Cryo-EM maps and models of the DUF4297-HerA complex with the N-terminal DUF4297 of the upper layer DUF4297 resolved. a Superimposition of cryo-EM maps of class 1 and class 2 to illustrate the densities of the N-terminal DUF4297 domain. **b** Superimposition of cryo-EM maps of class 1 and class 2 to illustrate different configurations of the linker between the N-terminal DUF4297 domain and CTD. **c** Superimposition of local refined maps of class 1 and class 2. **d** Composite model of class 1. **e** Composite model of class 2. **f** A B-form dsDNA is modeled into the central pore in the DUF4297-HerA complex.
