# Supplementary material for: Molecular and structural basis of an ATPase-nuclease dual-enzyme anti-phage defense complex
Source: Cell Res. 2024 Jun 4;34(8):545–55. doi: 10.1038/s41422-024-00981-w (PMC11291478; doi:10.1038/s41422-024-00981-w)
Supplement: Supplementary file 8 — Supplementary information, Fig. S8 [file 41422_2024_981_MOESM8_ESM.pdf]

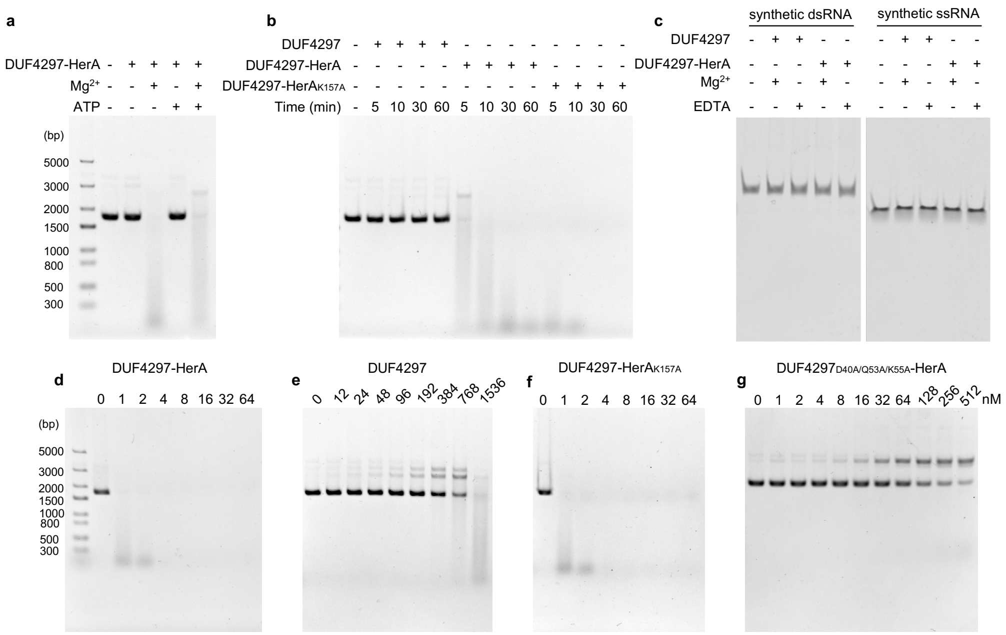

Supplementary information Figure S8. Nuclease activity of DUF4297-HerA complex and mutants, and DUF4297 alone. a Agarose gel analysis of DUF4297-HerA nuclease activity in the presence of cofactors. **b** Agarose gel analysis of DUF4297-HerA, DUF4297-HerA$_{K157A,}$ or DUF4297 alone nuclease activity at different reaction times. **c** Agarose gel analysis of DUF4297-HerA or DUF4297 alone nuclease activity on RNA substrates. **d-f** Agarose gel analysis of DUF4297-HerA (**d**), DUF4297 alone (**e**), or DUF4297-HerA$_{K157A}$ (**f**) nuclease activity at different protein concentrations. **g** Agarose gel analysis of DUF4297$_{D40A/Q53A/K55A}$-HerA nuclease activity at different protein concentrations.
