# Supplementary material for: Molecular and structural basis of an ATPase-nuclease dual-enzyme anti-phage defense complex
Source: Cell Res. 2024 Jun 4;34(8):545–55. doi: 10.1038/s41422-024-00981-w (PMC11291478; doi:10.1038/s41422-024-00981-w)
Supplement: Supplementary file 9 — Supplementary information, Fig. S9 [file 41422_2024_981_MOESM9_ESM.pdf]

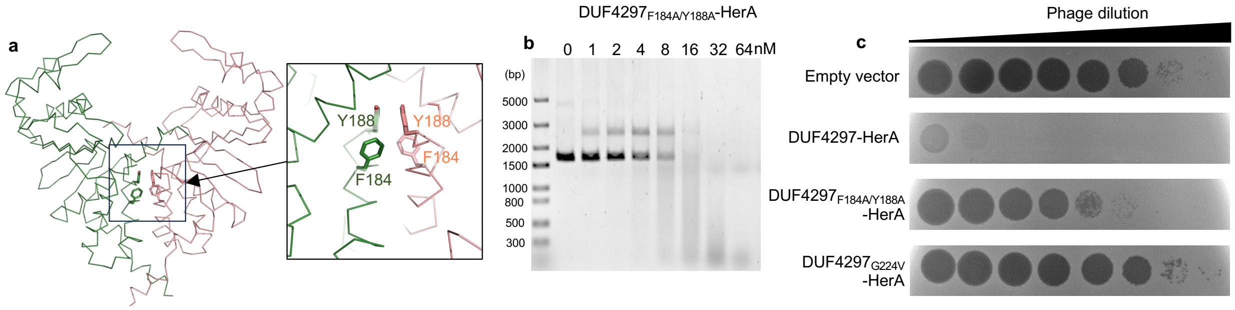

Supplementary information Figure S9. Perturbation of the N-terminal DUF4297 hydrophobic core reduced the nuclease activity and anti-phage ability. a Structural illustration of the N-terminal DUF4297 hydrophobic core shows the mutated residues as sticks. **b** Agarose gel analysis of DUF4297$_{F184A/Y188A}$-HerA nuclease activity at different protein concentrations. **c** Plaques of phage λ on cells expressing empty vector, WT DUF4297-HerA, and indicated mutants. 10-fold serial dilutions of the phage lysate were dropped on the plates.
