# Supplementary material for: Molecular and structural basis of an ATPase-nuclease dual-enzyme anti-phage defense complex
Source: Cell Res. 2024 Jun 4;34(8):545–55. doi: 10.1038/s41422-024-00981-w (PMC11291478; doi:10.1038/s41422-024-00981-w)
Supplement: Supplementary file 10 — Supplementary information, Fig. S10 [file 41422_2024_981_MOESM10_ESM.pdf]

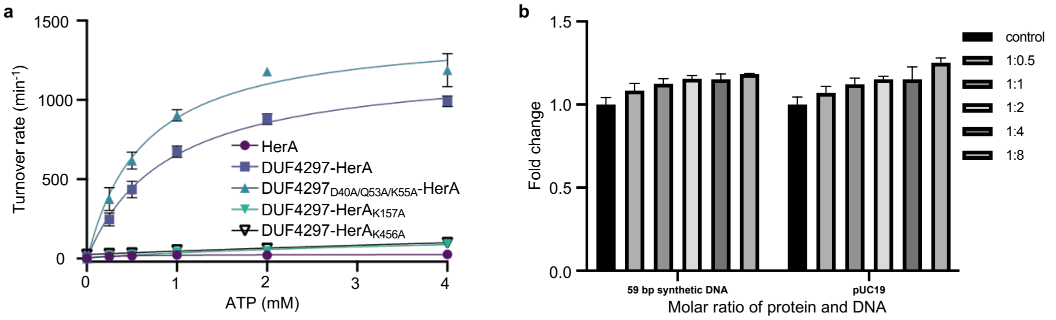

Supplementary information Figure S10. ATP activities of HerA, DUF4297-HerA complex and mutants. **a** ATP hydrolysis by DUF4297-HerA complex, DUF4297-HerA mutant complex or HerA. **b** ATPase activity of $DUF4297_{D40A/Q53A/K55A}$-HerA complex stimulated by synthetic DNA or pUC19 plasmid DNA.
