# Supplementary material for: Molecular and structural basis of an ATPase-nuclease dual-enzyme anti-phage defense complex
Source: Cell Res. 2024 Jun 4;34(8):545–55. doi: 10.1038/s41422-024-00981-w (PMC11291478; doi:10.1038/s41422-024-00981-w)
Supplement: Supplementary file 11 — Supplementary information, Fig. S11 [file 41422_2024_981_MOESM11_ESM.pdf]

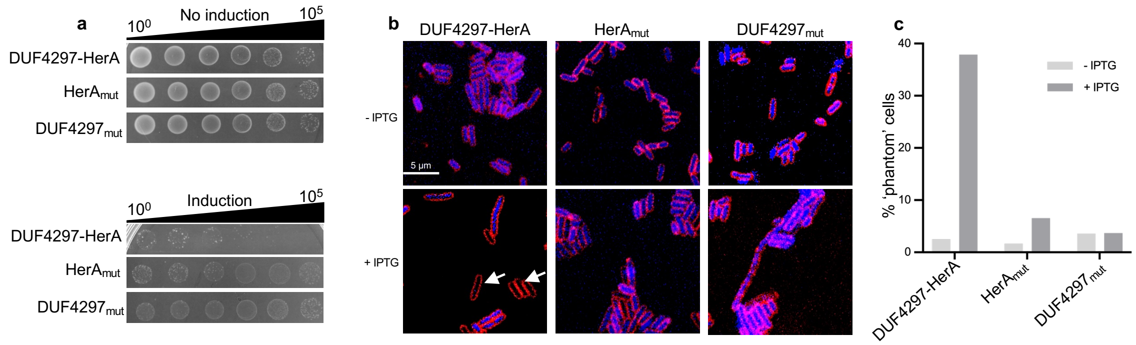

Supplementary information Figure S11. Consequences of DUF4297-HerA overexpression in *E. coli*. **a** Survival status of *E. coli* cells overexpressing DUF4297-HerA or mutants. **b** Representative images of cells overexpressing DUF4297-HerA or mutants. Cell membranes were stained with FM4-64 (red), and DNA was stained with DAPI (blue). White arrows indicate the 'phantom' cells devoid of both phage and host DNA. **c** Quantification of the percentage of 'phantom' cells in Figure **b**.
