# Supplementary material for: Molecular and structural basis of an ATPase-nuclease dual-enzyme anti-phage defense complex
Source: Cell Res. 2024 Jun 4;34(8):545–55. doi: 10.1038/s41422-024-00981-w (PMC11291478; doi:10.1038/s41422-024-00981-w)
Supplement: Supplementary file 12 — Supplementary information, Table S1 [file 41422_2024_981_MOESM12_ESM.pdf]

Supplementary information Table S1. Cryo-EM data collection, refinement, and validation statistics

	HerA (EMD-38203) (PDB 8XAU)	DUF4297-HerA (EMDB-38204) (PDB 8XAV)	DUF4297-HerA-AMPPNP-DNA state 1 (EMDB-38205) (PDB 8XAW)	DUF4297-HerA-AMPPNP-DNA state 2 (EMDB-38206) (PDB 8XAX)	DUF4297$_{Q53A/K55A}$-HerA-ATPγS-DNA (EMDB-38207) (PDB 8XAY)
**Data collection and processing**					
Magnification	50,000	50,000	50,000	50,000	50,000
Voltage (kV)	300	300	300	300	300
Electron exposure (e–/Å^2)	40	40	40	40	40
Defocus range (μm)	-0.5 to -2.5	-0.5 to -2.5	-0.5 to -2.5	-0.5 to -2.5	-0.5 to -2.5
Pixel size (Å)	0.95	0.95	0.95	0.95	0.95
Symmetry imposed	C1	C2	C1	C1	C1
Initial particle images (no.)	2,541,387	809,422	1,375,531	1,375,531	740,397
Final particle images (no.)	95,855	290,611	365,976	157,139	175,703
Map resolution (Å)<br>FSC threshold	3.14<br>0.143	2.87<br>0.143	2.73<br>0.143	2.92<br>0.143	2.81<br>0.143
Map resolution range (Å)	3.0-5.0	2.5-4.5	2.5-4.5	2.5-4.5	2.5-4.5
**Refinement**					
Initial model used (PDB code)	AlphaFold2	AlphaFold2	This study	This study	This study
Model resolution (Å)<br>FSC threshold	3.36<br>0.5	3.07<br>0.5	2.99<br>0.5	3.16<br>0.5	3.05<br>0.5
Model resolution range (Å)	3.4	3.10	3.00	3.20	3.10
Map sharpening *B* factor (Å^2)	-101.6	-102.1	-88.5	-82.9	-81.6
Model composition<br>Non-hydrogen atoms<br>Protein residues<br>Ligands<br>Nucleotide	 26,640<br>3,331<br>0<br>0	 48,264<br>5,886<br>0<br>0	 48,983<br>5,887<br>12<br>26	 48,954<br>5,887<br>9<br>26	 48,995<br>5,887<br>12<br>26
B factors (Å^2)<br>Protein<br>Ligand<br>Nucleotide	 61.96<br>0<br>0	 116.54<br>0<br>0	 16.25<br>12.93<br>13.42	 41.44<br>37.00<br>33.22	 30.44<br>18.07<br>22.90
R.m.s. deviations<br>Bond lengths (Å)<br>Bond angles (°)	 0.005<br>0.805	 0.004<br>0.838	 0.004<br>0.778	 0.004<br>0.785	 0.005<br>0.814
Validation<br>MolProbity score<br>Clashscore<br>Poor rotamers (%)	 1.68<br>7.73<br>0.43	 1.59<br>7.44<br>0.55	 1.53<br>6.68<br>0.60	 1.60<br>6.99<br>0.79	 1.49<br>6.44<br>0.66
Ramachandran plot<br>Favored (%)<br>Allowed (%)<br>Disallowed (%)	 96.35<br>3.65<br>0	 96.94<br>3.06<br>0	 97.09<br>2.91<br>0	 96.63<br>3.35<br>0.02	 97.32<br>2.67<br>0.02
